# Effectiveness of a Fully Impregnated Hydroxyapatite Polyetheretherketone Cage on Fusion in Anterior Cervical Spine Surgery

**DOI:** 10.7759/cureus.17457

**Published:** 2021-08-26

**Authors:** Kingsley R Chin, Nishant N Gohel, Daniel M Aloise, Jason A Seale, Deepak K Pandey, Fabio J Pencle

**Affiliations:** 1 Orthopedics, Florida International University, Miami, USA; 2 Orthopedics, Less Exposure Surgery (LES) Clinic, Hollywood, USA; 3 Faculty of Science and Sports, University of Technology, Kingston, JAM; 4 Orthopedic Surgery, Florida International University, Herbert Wertheim College of Medicine, Miami, USA; 5 Orthopedics, Florida International University, Herbert Wertheim College of Medicine, Miami, USA; 6 Orthopedics, Less Exposure Surgery (LES) Society, Hollywood, USA

**Keywords:** anterior cervical discectomy fusion, hydroxyapatite polyetheretherketone, degenerative disc disease, outcomes, less exposure surgery, cervical spine disorders

## Abstract

Introduction

Anterior cervical discectomy and fusion (ACDF) is the gold standard for the treatment of cervical spondylosis. However, new techniques, technologies, and improved implants have aided surgeons in reducing operative time with enhanced patient outcomes. Impregnated hydroxyapatite polyetheretherketone (HA PEEK) cages (Arena-C HA®, LESspine Inc. Malden, MA) are one such option that has aimed to increase the fusion rate. The authors herein aimed to assess the use of HA PEEK interbody cages by looking at outcomes, complications, and radiographic fusion.

Methods

The medical records of 41 consecutive patients undergoing single-level ACDF with impregnated HA PEEK cages (group 1) were compared to the control group of 47 patients who had single-level ACDF without impregnated HA PEEK cages (group 2). Outcomes assessed included Visual Analog Scale (VAS) neck, Neck Disability Index (NDI) scores, radiographic fusion, and complication rates.

Results

Of the 41 patients in group 1 (HA PEEK), 48% were female population with a mean age of 58.5+/- 1.7 years and BMI 29.7+/-1.2 kg/m^2^. Of the 47 patients in group 2 (non-HA PEEK), 53% were female with a mean age of 54.3+/- 1.2 years and BMI 27.8+/-0.8 kg/m^2^. Using t-test, there was a statistically significant intergroup difference in two-year VAS neck and NDI scores, p=0.007, and p=0.001, respectively. Radiographic fusion occurred as early as three months in the HA PEEK group.

Conclusions

This study has demonstrated the equivalence of impregnated HA PEEK cages in single-level ACDF. Significant improvements were seen in VAS and NDI scores in the HA PEEK group. There was no incidence of heterotopic bone formation or reaction to HA PEEK cages. Additionally, a trend toward fusion was seen in HA PEEK patients as early as three to five months compared to seven to eight months for the ACDF group. We conclude that HA PEEK cages can be safely placed with excellent outcomes. However, further studies are required to look at added benefits.

## Introduction

The improvement of cervical spine surgery continues to be pushed with newer technology that ultimately improves patient outcomes. Anterior cervical discectomy and fusion (ACDF) is the standard for treating cervical spondylosis, particularly degenerative disc disease. While studies have demonstrated excellent results in multilevel disease, for both inpatient and outpatient surgery, the introduction of hydroxyapatite has challenged this standard by improving upon ACDF [1-11]. Hydroxyapatite (HA) is an osteoconductive agent that aims to increase the fusion rate and improve patient outcomes in a shorter timeframe [12,13].

In conjunction with polyetheretherketone (PEEK) cages (Arena-C HA®, LESspine Inc., Malden, MA), which have biomechanical properties comparable to that of native bone and therefore fewer complications, impregnated HA PEEK cages stand to add value to current treatment methods [14]. Studies have demonstrated the efficacy of impregnated HA PEEK cages in the in vitro setting [15].

This study aimed to evaluate the clinical and radiographic outcomes with HA PEEK in anterior cervical fusion and possible complications. 

## Materials and methods

**Figure 1:**
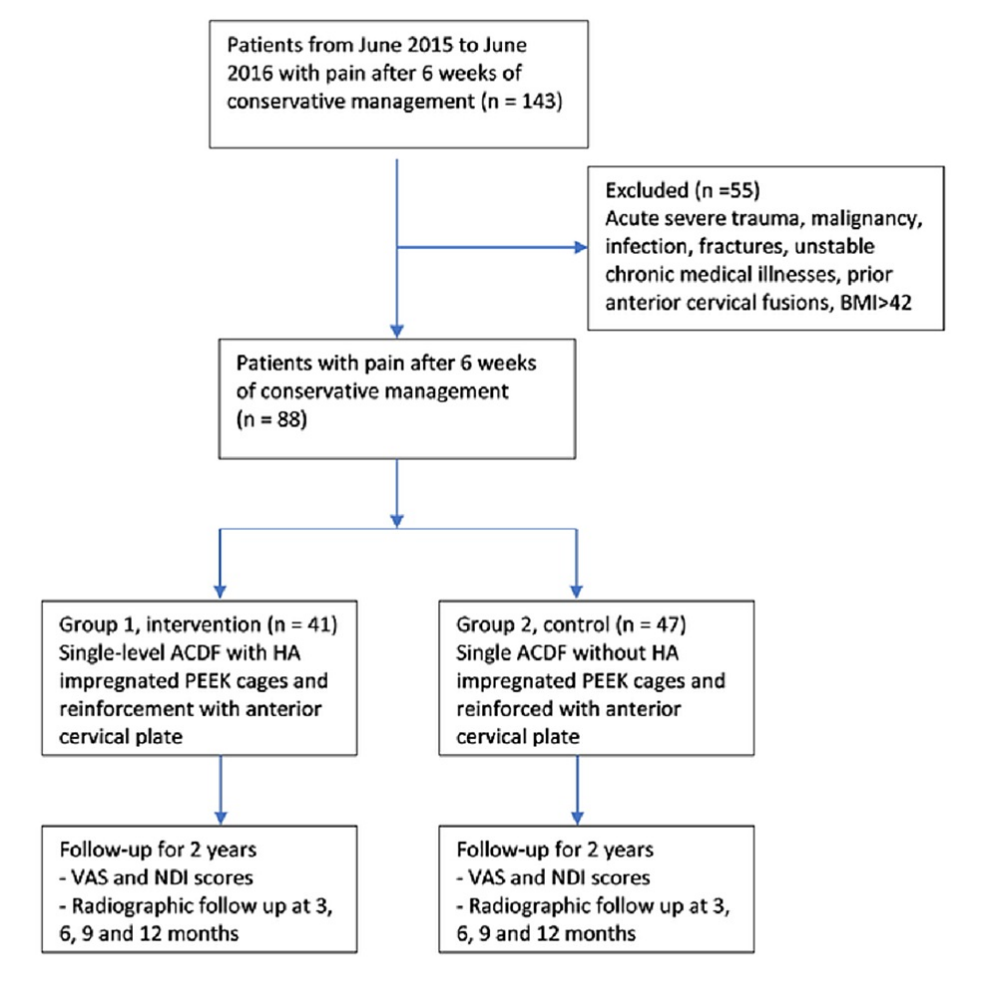
Flow chart showing inclusion and exclusion criteria. VAS: Visual Analog Score; NDI: Neck Disability Index; BMI: body mass index; ACDF: anterior cervical discectomy and fusion; HA: hydroxyapatite; PEEK: polyetheretherketone

This retrospective study of prospectively collected data from 2015 to 2016 had a total of 88 patients. Patients were enrolled from June 2015 to June 2016 and were followed for a minimum of two years. Two groups were created based on the type of implant choice by the surgeon. A total of 41 patients assigned to group 1 had single-level anterior cervical discectomy and fusion with impregnated HA PEEK cages; fusion was reinforced with an anterior cervical plate (ACP) (Inset®, LESspine Inc., Malden, MA). Our control group, group 2, included 47 consecutive patients who underwent single-level ACDF without impregnated HA PEEK; fusion was reinforced with an anterior cervical plate (ACP). IRB approval was obtained for the patients involved in the study as part of a cohort of anterior cervical surgery patients. All patients had signed consent to participate in the research study. Patients considered for the surgery were those who failed conservative management of pain for at least six weeks. Indications for ACDF surgery included cervical spondylosis, stenosing herniated discs, degenerative disc disease with instability and facet arthritis, tropism, or facetogenic pain. Acute severe trauma, malignancy, infection, fractures, unstable chronic medical illnesses, prior anterior cervical fusions, and BMI >42 were the exclusion criteria for surgery [16]. Figure 1 demonstrates inclusion and exclusion criteria. 

Surgical technique

Signed consent was obtained for the procedure and general anesthesia; patients were prepped and draped under sterile conditions before the surgery. For the procedure, a modified approach to the standard Smith-Robinson operative technique was used [17]. A 1.5-inch midline anterior cervical incision was done to achieve the surgical exposure of the desired vertebral level. Subcutaneous dissection was performed for adequate mobilization to tissue. Pituitary rongeurs, curette, and burr drill were used to remove the affected disc, the posterior longitudinal ligament was retained in situ [18,19]. ACDF was performed after discectomy; the appropriately sized PEEK cage with demineralized bone matrix (DBM) was inserted. The smallest sized ACP was placed by predrilling holes with a guide. Once hemostasis was achieved, a Penrose drain was placed above the implant, brought through the incision, and secured with a sterile safety pin. This was done in all patients for wound drainage to prevent postoperative hematoma development at home. 

Discharge and follow-up

Outpatient postoperative instructions were provided to patients and caregivers after being discharged. Published discharge criteria were used as a protocol [16,20]. Potential complications were explained to the patient by a member of the outpatient surgery team. After 24-48 hours, removing the Penrose drain by a medical staff member was observed once there was no active drainage. Outcomes reported by the patient included Visual Analogue Scales (VAS) for pain and Neck Disability Index (NDI) for disability scores. Analysis comparison between groups was performed both preoperatively and at the final two-year follow-up period.

Statistical analysis

Statistical analysis using SPSS version 22 (Armonk, NY: IBM Corp.) was performed to determine significance using a p-value of <0.05. T-test was used to calculate the mean and standard deviation. Power analysis was performed, demonstrating that an adequate sample size of 30 patients per group was necessary to verify statistical differences, with a power = 0.8 and alpha = 0.05.

## Results

Of the 41 patients in group 1 (HA PEEK ACDF), 48% were female with a mean age of 58.5+/- 1.7 years and BMI 29.7+/-1.2 kg/m^2^. Of the 47 patients in group 2 (ACDF), 53% were female with a mean age of 54.3+/- 1.2 years and BMI 27.8+/-0.8 kg/m^2^. No statistical differences in gender, age, and BMI were found between the groups, p=0.831, 0.078, and 0.084, respectively. Demographics are summarized in Table 1, including pathological levels and chief complaint (indication for operation).

**Table 1:** Cohort demographics with pathological levels and chief complaint. BMI: body mass index; HA: hydroxyapatite; PEEK: polyetheretherketone

Variable	HA PEEK	Non-HA PEEK
Age (years)	58.5+/-1.7	54.3+/-1.2
BMI (kg/m^2­­^)	29.7+/-1.2	27.8+/-0.8
Male	20	22
Female	21	25
Pathological level		
C3-4	6	7
C4-5	11	9
C5-6	14	15
C6-7	7	11
C7-T1	3	5
Diagnosis		
Herniated disc	8	10
Degenerative disc disease	15	13
Spondylosis (chronic pain)	4	9
Myelopathy	7	11
Radiculopathy	7	4

**Figure 2:**
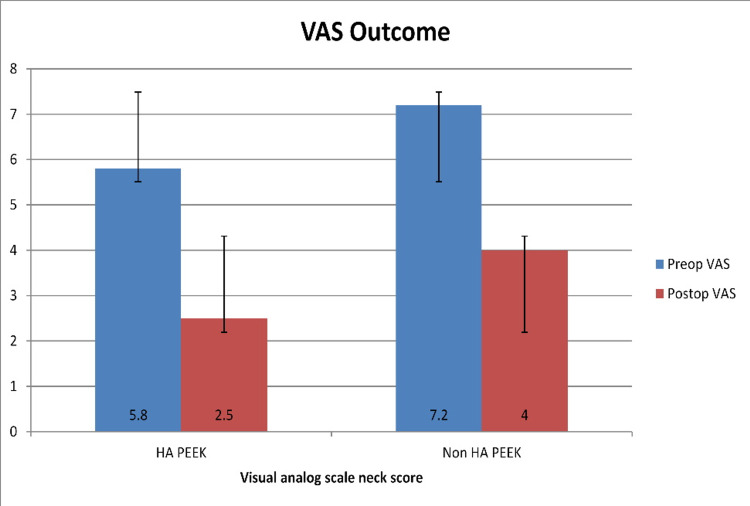
Bar chart showing preoperative and postoperative mean VAS neck scores with standard deviations. VAS: Visual Analog Score; HA: hydroxyapatite; PEEK: polyetheretherketone

**Figure 3:**
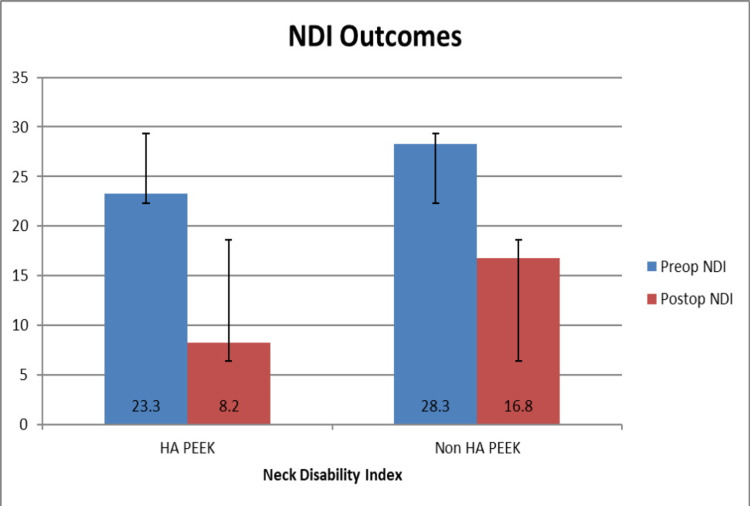
Bar chart showing preoperative and postoperative mean NDI scores with standard deviation. NDI: Neck Disability Index; HA: hydroxyapatite; PEEK: polyetheretherketone

There was no significant difference between preoperative VAS neck and NDI scores between groups 1 and 2, p=0.548, 0.187. Analysis of follow-up at the two years demonstrated: group 1 mean preoperative VAS neck scores improved from 5.8+/-0.3 to 2.5+/-0.3 at two-year follow-up, p<0.001. Preoperative mean NDI score decreased from 23.3+/-1.1 to 8.2+/-1.0 at two-year follow-up, p<0.001. Group 2 mean preoperative VAS neck scores improved from 7.2+/-0.3 to 4.0+/-0.2 at two-year follow-up, p=0.001. Preoperative mean NDI reduced from 28.2+/-3.4 to 16.8+/-1.3 at two-year follow-up, p=0.001. An overall improvement in VAS neck and NDI scores is shown in Figure 2 and Figure 3, respectively. Comparing postoperative outcomes between groups 1 and 2 showed statistically significant differences in VAS neck and NDI scores, p=0.007, and p=0.001. 

**Figure 4:**
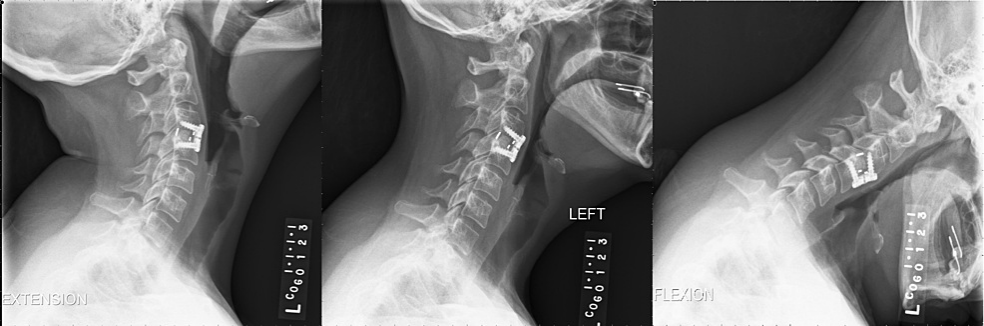
X-ray demonstrating bridging bone in HA PEEK group. HA: hydroxyapatite; PEEK: polyetheretherketone

**Figure 5:**
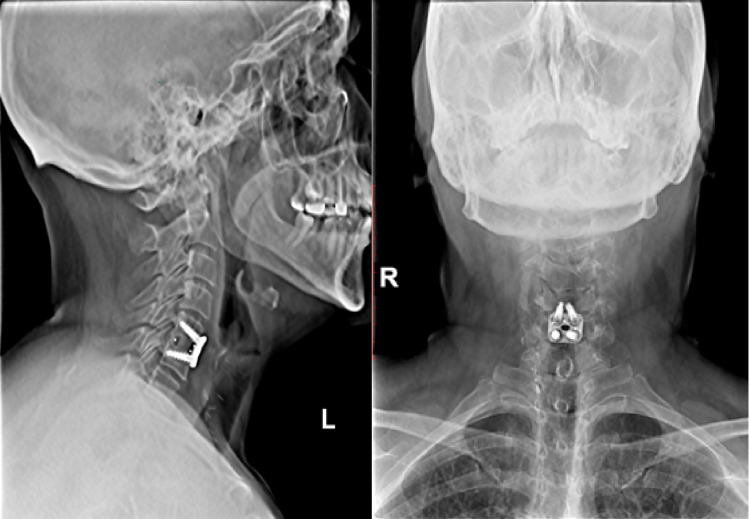
X-ray showing bridging bone in non-HA PEEK group. HA: hydroxyapatite; PEEK: polyetheretherketone

Postoperative radiographs were performed at three, six, nine, and 12 months. Fusion was defined as <1 mm of motion on plain radiographs, including flexion and extension views [21]. Fusion rate was faster in the HA PEEK group, with fusion noted as early as three months and up to five months (Figure 4) compared to seven to eight months in the non-HA PEEK group (Figure 5).

The main postoperative complaint of postoperative dysphagia was defined as discomfort or difficulty swallowing that was not present before the surgery. The severity of postoperative dysphagia was assessed using the Bazaz-Yoo dysphagia severity scale (mild, moderate, and severe) over the initial three-month postoperative period [22]. This complication occurred in both groups with mild severity and transiently, with the longest period of six weeks in two patients who received HA PEEK ACDF, compared to six patients who received only ACDF, p=0.275. 

## Discussion

We aimed to assess the outcomes of HA PEEK ACDF. Impregnated HA Arena-C cage is the first cervical interbody manufactured by Invibio using PEEK-Optima® HA enhanced. The FDA cleared the Arena-C HA device in October 2014. PEEK OPTIMA® is a polyetheretherketone (PEEK) impregnated with hydroxyapatite (HA), a naturally occurring compound in human bone. PEEK-OPTIMA LT120HA (PEEK-OPTIMA HA[i] Enhanced) is a high-performance, implant-grade polymer manufactured by Invibio Ltd. (Lancashire, UK). The compound contains 80% PEEK-OPTIMA LT1 and 20% calcium hydroxyapatite (HA) [23]. In vivo studies have demonstrated the HA PEEK creates a more favorable environment for fusion by improving the bone-implant interface. [15]

The present study shows significant improvement in postoperative outcomes in both groups; intergroup significance was noted at the final two-year follow-up for VAS neck and NDI scores. There were no major complications in this study, which implemented appropriate postoperative follow-up over 12 months. Dysphagia was the most common complication noted in both groups. However, we demonstrated no significant difference between groups. 

In a study by Mashhadinezhad et al., 112 patients were implanted with PEEK cages packed with an autologous graft taken from the iliac crest of the patients, and 124 patients were implanted with PEEK cages filled with hydroxyapatite (HA) granules [24]. These patients were followed up at three months and 12 months. The investigators showed that the formation of bony bridges at the three months follow-up was higher in the autograft versus the granule group (16.6% vs. 8%). However, they did not find a difference between these groups at the 12-month follow-up assessment [24]. Our results demonstrate a clinically relevant faster rate of fusion, based on postoperative plain radiographs, including flexion and extension views in the HA PEEK ACDF group. Furthermore, studies have shown enhanced bone growth in cortical and cancellous sites by incorporating HA into PEEK compared with PEEK alone [15,25], where investigators have reported the presence of a non-reactive fibrous tissue interface [26,27]. In addition, the HA at the surface of PEEK provides an osteoconductive surface, which supports bone apposition, thus improving rates of fusion clinically [25].

Strengths and limitations

The robustness of this study is the sample size and the clinical and radiographic assessment of outcomes, a univariate statistical analysis was conducted. The study was a clinical cohort study with the aim to assess outcomes. The outcomes assessed include patient factors (e.g., age, sex, BMI), VAS, NDI scores, fusion rate, and complications. The limitations of this study were a retrospective review of prospectively collected data from two cohort populations. We acknowledge the variations of diagnosis in each group; however, each cohort had a similar number of patients with each diagnosis. Because the data were collected prospectively, the number of patients required in each group to achieve statistical significance was determined based on power analysis. There was no randomization for the use of an implant. 

## Conclusions

This study has demonstrated the equivalence of impregnated HA PEEK cages in single-level ACDF. Statistically, significant improvement was seen in the HA PEEK group in VAS and NDI scores. There was no incidence of heterotopic bone formation or reaction to HA PEEK cages, and the trend toward fusion was seen in HA PEEK patients as early as three to five months, as compared to beginning at seven to eight months in the ACDF group. We conclude that HA PEEK cages can be safely implanted with improved outcomes compared to the current standard of treatment in the form of PEEK cages alone. This study adds to the literature for clinical studies performed using HA PEEK. Further studies are encouraged to assess for additional benefits of the HA PEEK cages.
